# The Treatment of Carbuncle

**Published:** 1882-07-20

**Authors:** S. Baruch

**Affiliations:** N. Y. City, Ex-President and Honorary Member S. C. Medical Association


### THE TREATMENT OF CARBUNCLE.

BY S. BARUCH, M.D., N. Y. CITY,

Ex-President and Honorary Member S. C. Medical Association.

There are few diseases which have been subjected to more varied methods of treatment than carbuncle. The able essay by Dr. McF. Gaston on the latter subject, which appeared in the September number of your Journal, refers to a certain mode of management so nearly akin to that which I regard as the most successful, that I am led to write this brief, practical communication.

The crucial incision, with its various modifications, so glowingly advocated by Syme, Collis and others, has had its day and, together with depleting measures, has passed into desuetude. Regarded as a disorder due to or accompanied by conditions of debility, there is now little difference of opinion with reference to the admissibility, nay, the necessity, of general and local supporting measures.

In the course of an experience of twenty years, the following treatment of carbuncle has gradually developed into a successful mode of management.

Beginning with the classical crucial incision, I did not fail to recognize its great utility in the amelioration of pain. I have no doubt that I have transposed patients from the throbbing agony so characteristic of carbuncle to a condition of great comfort, by freely laying open the tense and brawny tissues. But the relief was temporary, the disease was not checked, the loss of blood from the turgid vessels occasionally prostrated the patient, and altogether the result offered an analogy to the depleting treatment formerly adopted in pneumonia.

In fact, the analogy between the management of the latter disease and the management of carbuncle struck me, as time passed on. Just as Bennet inveighed against bloodletting in pneumonia, and with Dietl introduced the expectant plan, so did Paget eloquently plead for an expectant treatment of carbuncle.

But the latter progressed step by step, until now it presents the same modified form, bearing the impress of careful study and attention to detail which has made the former a more manageable disease.

In August, 1863, Dr. Prichard published in the British Medical Journal an article in which he suggested the value of collodion combined with iodine and iod. potass., as a support, with absorbent action in carbuncle. Pressure by strips of adhesive plaster has been advocated by some, but in my experience the collodion which I have long used without iodine is superior, inasmuch as it adapts itself to the uneven surface of the tumor, and by gradual compression supports the paretic coats of the vessels, aids in expelling the liquid detritus, and affords comfort to the patient which is remarkable. I have often noticed after the crucial incision, liquid and semi-solid matter oozing from the exposed surface, so soon as the collodion began to contract. The hemorrhage, too, was moderated, and the relief from tension, inaugurated by the incision, became more lasting.

For the next improvement in the treatment of carbuncle we are also indebted to an Englishman. In Braithwaite's Retrospect of July, 1871, Dr. Murray recommended the use of potassa fusa in lieu of the incision. This caustic had been formerly recommended for the purpose of avoiding the pain of the incision and facilitating the sloughing. But Dr. Murray presented it for a different purpose, viz: to abort the carbuncle, and I can testify to the success of the "crucial scoring" of the unbroken surface of a beginning carbuncle. When a patient presents himself in the incipient stage, ere vesicles are fully formed, I apply the solid potassa fusa in two crucial lines, gently rubbed into the tense integument as far as the dusky discoloration extends. Even when the vesicles are formed, but before sloughing has commenced, this crucial scoring will be useful; but the pain resulting from it in these cases has deterred me from frequently resorting to it. Collodion is next brushed in three or four successive coats upon the diseased surface, excluding the crucial lines. A light flaxseed poultice, warm and soft, is applied over the whole, and in many instances the relief is marvellous. Pain ceases at once. In a few days, the central portion of the tumor sinks, a thin tegumentary slough separates and the carbuncle seems to melt away.

In more advanced cases a different course is pursued. The recommendations of Dr. Eade, of London, (who regards carbuncle as a parasitie disease) to introduce carbolic acid within the tumor, has proven a boon to me in these trying cases. The pure carbolic acid liquefied and held in liquid form by a few drops of glycerine, is frequently carried by means of a camel's hair brush into every open point, after the slough channel has been cleansed by a pointed tent of linen. This application must be made thoroughly, but very gently. If properly done the pain will not be severe, and its daily repetition, which is necessary, will not be dreaded by the patient. After this application, collodion is freely brushed in three or more successful coats, over the entire diseased surface, extending a few lines beyond the outline even. A doubled piece of linen, having a central opening to admit the sloughing portion of the carbuncle, is now laid upon the collodion-covered surface. A light flax-seed poultice is placed over the former, and renewed several times a day. Tincture of iron and quinine, milk, any other nutriment the patient can be induced to take, are freely administered, anodynes are prescribed whenever necessary, with a view to allay pain and prevent loss of sleep. The latter are rarely needed after the first day. If the case progresses favorably, the collodion dressing is continued daily, but the carbolic acid may be omitted every other day. Patient is urged to go out into the open air, or to be carried out when unable to move without pain.

Under this management carbuncle may be carried to its termination, with a minimum of pain to the patient and a maximum of satisfaction to the surgeon.—*Ame. Med. Bi- Weekly.*

